# Middle ear status – structure, function and pathology: A scoping review on middle ear status of COVID-19 positive patients

**DOI:** 10.4102/sajcd.v69i2.910

**Published:** 2022-07-25

**Authors:** Ben Sebothoma, Katijah Khoza-Shangase

**Affiliations:** 1 Department of Speech Pathology and Audiology, Faculty of Humanities, University of the Witwatersrand, Johannesburg, South Africa

**Keywords:** COVID-19, middle ear function, scoping review, pathology, symptoms

## Abstract

**Background:**

The coronavirus disease 2019 (COVID-19) pandemic is the latest public health emergency that has presented challenges globally. Limited evidence exists on the association between COVID-19 and middle ear pathologies, regardless of the respiratory nature of some of the core symptoms.

**Objective:**

This scoping review aimed at exploring evidence on the effects of COVID-19 on middle ear functioning as part of symptom mapping and preventive planning for ear and hearing care.

**Method:**

Electronic bibliographic databases, including Medline, ProQuest, PubMed, Science Direct, ERIC and Scopus, were searched to identify peer reviewed publications, published in English, between December 2019 and January 2022, related to the effects of COVID-19 on middle ear functioning. The keywords used as MeSH terms included ‘middle ear pathology’, ‘middle ear disorder’, ‘otitis media’, ‘hearing loss’, ‘hearing impairment’, ‘audiology’ and ‘COVID-19’ or ‘coronavirus’.

**Results:**

From eight studies that met the inclusion criteria, the findings revealed that middle ear pathologies occur in this population, with the occurrence ranging from 1.15% to 75%. Tympanic membrane structural changes, otitis media and conductive hearing loss (CHL) were commonly reported. The current findings must be interpreted with caution given that most of the studies reviewed had extremely small sample sizes or were case studies or series, thus limiting generalisability.

**Conclusion:**

The findings highlight the value of strategic research planning to collate data during pandemics, ensuring that future studies use appropriate and well-designed methodologies. Trends and patterns of middle ear pathologies in this population must also be established to determine the need for periodic monitoring.

## Introduction

The latest pandemic (coronavirus disease 2019[COVID-19]) that ‘caught the world by surprise’ (Clark et al., 2020) is caused by severe acute respiratory syndrome coronavirus-2 (SARS-CoV-2) that leads to severe respiratory diseases. Since the discovery of COVID-19 pandemic, the number of cases has been increasing rapidly and exponentially, spreading to multiple countries around the world (Chowdhurry & Oommen, 2020). As of 27 January 2022, the World Health Organization (WHO) has reported that there are approximately 360 million confirmed cases of COVID-19, with approximately 5.6 million deaths reported globally (World Health Organization, 2022). The USA has the highest number of COVID-19 infections, reaching 100 million, with African countries having the least number of cases and South Africa leading in these numbers (World Health Organization, 2021).

Since the advent of COVID-19, there has been a global effort to understand and establish its clinical manifestations to formulate preventive measures, as well as treatment plans. Current evidence indicates that the clinical manifestations of COVID-19 are primarily respiratory in nature (Da Rosa Mesquita et al., 2020; Hu, Guo, Zhou, & Shi, 2021). Some patients infected with COVID-19 exhibit respiratory-related symptoms such as cough, fever, fatigue and myalgia (Zu et al., 2020), while other patients may remain asymptomatic (Parasher, 2021). The complexity of the prevention of the transmissibility of the virus seems to be because some patients remain asymptomatic (Huang et al., 2020), and the virus is not clearly detectable during this stage (Pan et al., 2020), thus making transmission difficult to detect and prevent.

While COVID-19 predominantly affects the respiratory system, auditory and vestibular difficulties such as vertigo have also been reported among individuals infected with the COVID-19 virus (Aljasser, Alkeridy, Munro, & Plack, 2021; Jafari, Kolb, & Mohajerani, 2021). A systematic review conducted by Almufarrij and Munro (2021) indicated that the prevalence of hearing loss is approximately 8%, with sensorineural hearing loss (SNHL) being the most common type of hearing loss. Kilic et al. (2020) posit that SNHL, which is predominantly sudden in nature, results from the effects of the virus on the cranial nerves, while Jafari et al. (2021) and Mustafa (2020) suspect that the SNHL results from the direct effects of COVID-19 infection on the Organ of Corti, stria vascularis and/or spiral ganglion. Given this evidence, although not significant, early ear and hearing pathology identification and intervention methods may need to be prioritised for patients infected with COVID-19. This should include implementation of preventive programmes such as hearing screening or/and monitoring programmes, as well as referral for COVID-19 testing where patients present with middle ear pathologies during this pandemic.

Given that COVID-19 appears to affect the auditory system, the need for hearing health professionals such as audiologists is very clear and crucial, particularly for early identification and intervention of auditory pathologies. Research has indicated that auditory pathologies such as middle ear pathologies, if not identified early, can have multiple impacts such as impaired processing of information and communication difficulties (Brennan-Jones et al., 2020; Kolo, Salisu, Yaro, & Nwaorgu, 2012), and overall negative effects on the affected individual’s quality of life (Reddy, Priyanka, Sreenath, & Reddy, 2015). Furthermore, untreated middle ear pathologies have been shown to cause life-threatening conditions such as brain abscess (Ibrahim, Cheang, & Nunez, 2010). Therefore, in the midst of COVID-19, which directly or indirectly affects the auditory system, audiologists should form part of the preventive and management care of patients infected with the virus.

While initial evidence indicates that the auditory system may also be impacted by COVID-19, limited research exists that reports on the effects of COVID-19 on the middle ear system. Given that coronaviruses affect the immune system (Bobcakova et al., 2021), the upper respiratory system (Weiss & Navas-Martin, 2005), which is often associated with the development of middle ear pathology, there is a need for further research in this area. An exploration of this area of research may provide an insight into the need for early identification and intervention, and resource allocation to prevent the long-term consequences of untreated middle ear pathologies resulting from COVID-19 and improve the quality of lives.

## Methodology

### Aim

This study aimed to review published evidence on the effects of COVID-19 on middle ear functioning.

### Research design

A scoping review was adopted for the purpose of this study (Levac, Colquhoun, & O’Brien, 2010). This review was deemed to be appropriate for this study as researchers intended to explore the literature and determine the type of evidence that exists on COVID-19 and middle ear function (Nelson & Gilbert, 2021). This scoping review was conducted according to the framework by Arksey and O’Malley (2005), where (1) the broad research question was identified, (2) the relevant publications were found, (3) the study was selected, (4) data charting was performed, and (5) the results were collated, summarised and reported.

### Research question

This current review was guided by the following broad question: what has been published about the effects of COVID-19 on middle ear function? This question is motivated by strategic planning needs for both COVID-19 spread prevention and early identification and management of middle ear pathologies, pathologies which are otherwise treatable and their effects reversible. This question is over and above establishing a link between COVID-19 and middle ear pathologies so that the COVID-19 disease symptomatology can be comprehensive.

### Data sources and strategy

The initial search was carried out in December 2021. A comprehensive search of papers that have reported on COVID-19 and middle ear pathologies was conducted using online databases, including Medline, ProQuest, PubMed, Science Direct, ERIC and Scopus. Published articles were included if they were published between December 2019 and January 2022 to coincide with the advent of COVID-19, if they reported on COVID-19 and middle ear function, and were published in English. The search terms that were used for this scoping review included ‘middle ear pathology’, ‘middle ear disorder’, ‘otitis media’, ‘hearing loss’, ‘hearing impairment’, ‘audiology’ and ‘COVID-19’ or ‘coronavirus’.

### Citation management

All citations were imported into the web-based bibliographic manager endnote, where duplicate citations were removed.

### Title and abstract relevance screening

Each researcher, using Arksey and O’Malley’s (2005) framework, independently screened the titles and abstracts of the articles retrieved through the database search. In the first phase, the titles of the articles were screened to determine whether they were relevant. If the title of the article seemed relevant, an abstract review was conducted in the second phase. If the article met the inclusion criteria, then a full manuscript review was conducted in the final phase. In cases where the article did not have an abstract, but the title seemed relevant, the article was included for full review in the third phase. The title, abstract and full article review were conducted by each researcher. An inter-rater agreement was calculated to determine the level of agreement for studies included and excluded for this review. A high inter-rater agreement of 94.3% was achieved between the reviewers, which indicates an almost perfect agreement (Viera & Garrett, 2005).

### Characterisation of included studies

After all the articles were screened, and agreement was made between the researchers on which articles were relevant for this review, the researchers developed an excel spreadsheet which was used to document pertinent details of each article. Table 1 depicts the important details that the researchers charted. Such details included the author’s name and date of publication, title of the publication, context from which the study was conducted, aim of the study, signs and symptoms of middle ear pathology, conclusion, and recommendations. Table 2 depicts the test procedure used in various studies. Data was charted by both researchers. In this phase, researchers maintained constant engagement to resolve any differences that may have been present.

**TABLE 1:** Details of study characteristics of each study included.

Author	Study title	Aim of study	Study design	Country	Signs and symptoms	Conclusion	Recommendations
Bhatta et al. (2021)	Study of Hearing Status in COVID-19 Patients: **A Multicentred review.**	To evaluate the hearing status of COVID-19 patients and compare with control group (pure tone audiogram and impedance audiometry of COVID-19 patients performed initially and at 3 months follow-up)	A Multicentred review.	Nepal and India	Aural symptoms were: aural fullness in 1.4%, hearing loss in 3.9%, and earache in 1.8% were present initially, resolved at 3 months follow-up. Impedance audiometry: type B and type C tympanograms in 5.1% and 1.15% ears, and out of these 64.7% and 40% improved at 3 months follow up, respectively. 3.2% with mild CHL, but no significant difference observed between the average air conduction and bone conduction of the COVID-19 patients and control group.	The COVID-19 infection may present with aural symptoms; however, it was concluded that there was no significant difference in the hearing status of the COVID-19 positive patients in comparison with the control group. The presence of some changes in the normal functioning of the eustachian tube and middle ear in the COVID-19 infection was also highlighted.	-
Boroujeni et al. (2021)	Acute otitis media and COVID-19 symptoms: a case report	To present a 39-year-old case of a female patient complaining of earache and hearing loss with no other COVID-19 symptoms.	Case report	Isfahan, Iran	Complained of sense of fullness, hearing loss in the left ear. Redness and bulging of TM noted. Type B tymp, absent reflexes and mild to mod-severe CHL	Acute otitis media can be the only symptom in the absence of common COVID-19 symptoms. Because COVID-19 has been identified as a known cause of upper respiratory infection, otitis media could be expected. Prolongation of patients’ nasopharyngeal and eustachian tube tissues’ oedema caused by possible COVID-19 induced disorders in the mucociliary and immune system performance leads to negative pressure, thus causing the middle ear to become susceptible to secondary viral and bacterial infections.	COVID-19 should also be suspected where the only observed symptom is earache with hearing loss.
Dharmarajan et al. (2021)	Hearing loss – a Camouflaged manifestation of COVID-19 infection	To assess the audiological profile among 100 mild to moderately affected COVID-19 individuals, so as to make a contribution to the emerging literature on otologic manifestations in COVID-19.	Case series	India	Otoscopic examination showed normal external auditory canal and tympanic membrane in 95 patients, 4 patients had retracted TM, and 2 had dull TM. 6 patients had CHL. 18 had a referred OAE	Early identification and intervention if required help to give a better quality of life to the patient	-
Enrique, Margarita, Ángel, Saturnino and Jesús (2021)	COVID-19 and severe ENT infections in paediatric patients. IS there a relationship?	To determine whether there is a relationship between COVID-19 and severe infections in the ear, nose, throat and deep cervical area (ENT) in paediatric populations	Retrospective observation study	Spain	Found a significant outbreak in the incidence of complicated mastoiditis and deep cervical infections with complications in the year 2020 (13 patients) linked to the COVID-19 pandemic.	The limitations in primary care due to a shortage of human resources in dealing with the pandemic’s initial onslaught and changes in help-seeking behaviour could explain increased complicated infections.	-
Fidan (2020)	New type of COVID-19 induced acute otitis media in adult.	To present an adult case with COVID-19 with otitis media, without any classical COVID-19 symptoms	Case report	Turkey	Presented with otalgia and tinnitus, Hyperaemia and bulging TM. Type B tymp and CHL in the right ear	COVID-19 can manifest itself with different findings, without the classic symptoms, and complete body examination is most important in the evaluation of patients	-
Maharaj, Bello Alvarez, Mungul, and Hari (2020)	Otologic dysfunction in patients with COVID-19: **A systematic review**	To describe otologic dysfunction in patients with the novel SARS-CoV-2.	Systematic review	N/A	There were 28 patients in total identified with the largest study comprising 20 patients. All patients presented with hearing loss *(mostly Sudden Sensorineural hearing loss, 2 cases of otitis media/CHL*, 27 of whom had audiometry. Three patients had associated vestibular symptoms (vertigo, otalgia, and tinnitus).	SARS-CoV-2 is a probable cause of middle ear infections and sensorineural hearing loss, secondary to spread of the novel virus into the middle ear and related neural structures. Currently, there is no evidence supporting the presence of SARS-CoV-2 in the middle ear, but findings of this review implicate the virus as a potential source of otologic disorders. Mechanism of hearing loss in this case was a middle ear effusion secondary to an ascending nasopharyngeal infection	-
Raad, Ghorbani, Mikaniki, Haseli and Karimi-Galougahi (2021)	Otitis media in coronavirus disease 2019: A case series	To assess the presence of otitis media in a series of patients with confirmed COVID-19 and ENT symptoms	Case series	Iran	The present case series includes eight patients who presented over a 2-month COVID-19 pandemic period. Six of the eight patients had otalgia, and seven had HL. Middle ear effusion was evident in six patients on otoscopic examination. Three patients had typical signs of acute otitis media, one had acute otitis media with TM perforation. Most patients had CHL	Otitis media should be considered a manifestation or associated symptom of COVID-19 during the current pandemic	Otitis media was the first manifestation of COVID-19 in some patients in this case series. Thus, it is recommended that during the current pandemic, the presence of otitis media should alert clinicians to the possibility of Covid-19
Wanna et al. (2021)	COVID-19 sampling from the middle ear and mastoid: A case report	To report the case of a recently hospitalised COVID-19 positive patient with a previous history of canal-wall down mastoidectomy for cholesteatoma who required mastoid cavity debridement	-	-	Patient present with aural fullness, and purulent otorrhea	More investigation is needed prior to making definitive recommendations on middle ear or mastoid manipulation in the COVID-19 positive patient	-

COVID-19, coronavirus disease 2019; CHL, conductive hearing loss; OAE, Otoacoustic emissions; ENT, Ear, Nose and Throat; SARS-CoV-2, severe acute respiratory syndrome coronavirus 2; HL, hearing loss.

**TABLE 2:** Details of the study test procedure for determining middle ear pathologies (MEP).

Author	Test procedure
Bhatta et al. (2021)	Case history, pure tones and impedance audiometry
Boroujeni et al. (2021)	Otorhinolaryngological examination, tympanometry and pure tone audiometry
Dharmarajan et al. (2021)	Clinical examination, pure tone audiometry and transient evoked otoacoustic emission
Enrique et al. (2021)	Retrospective analysis of the clinical history
Fidan (2020)	Otorhinolaryngological examination, tympanometry and pure tone audiometry
Maharaj et al. (2020)	Description of various studies’ results
Raad et al. (2021)	Otorhinolaryngological examination and case history
Wanna et al. (2020)	Otorhinolaryngological examination and case history

### Data summary and synthesis

**FIGURE 1:**
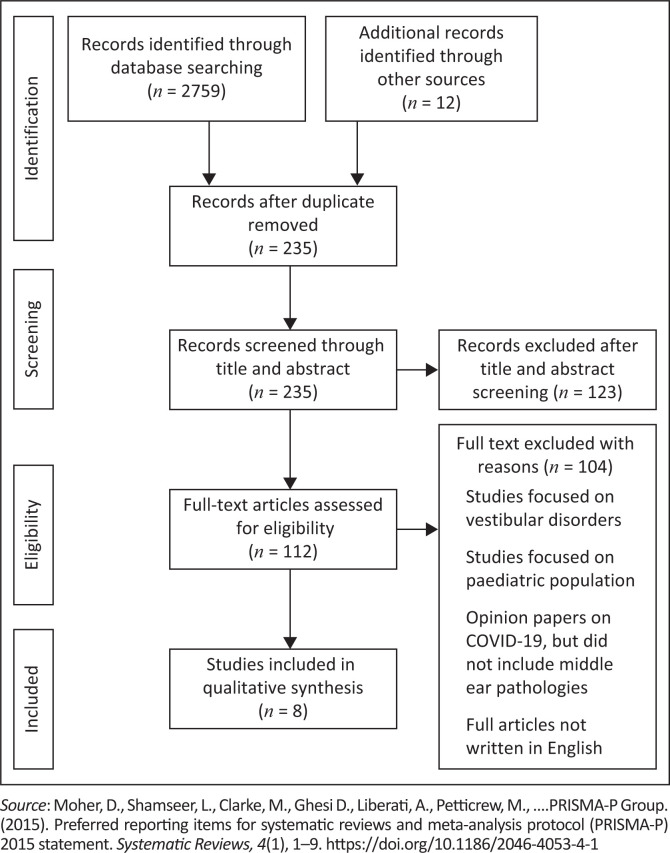
The PRISMA flow diagram describing the process of study selection.

All data were recorded in a Microsoft Excel 2016 (Microsoft Corporation, Redmond, WA, USA) spreadsheet for descriptive analysis, where thematic analysis following Braun and Clarke (2006) was conducted. The initial search yielded a total of 2771 articles as depicted in the Preferred Reporting Items for Systematic Reviews and Meta-Analyses (PRISMA) diagram (Figure 1) (Moher, Liberati, Tetzlaff, Altman, & The PRISMA Group, 2009). After removing duplicates from different database, 235 records remained, of which 32 articles were fully reviewed. Of these 32 articles, a further 25 articles were excluded as they did not provide any information on the link between COVID-19 and middle ear function or pathology. This resulted in eight articles included for analysis.

### Ethical considerations

This scoping review followed all ethical standards for a study that does not involve direct contact with human or animal participants. This entailed researchers engaging in reflexivity and observing informed subjectivity, adhering to audience-appropriate transparency, as well as informed selective inclusivity, as guided by Suri (2020).

## Results and discussion

As shown in Table 1, pertinent information from the studies that were analysed thematically were provided. A total of eight studies that were finally included for analysis comprised of a range of studies conducted in different contexts. Studies included in this scoping review were mainly case studies or case series, with the remaining studies consisting of a multicentre, retrospective observation and a systematic review. Among the studies, 4 were conducted in low- and middle-income countries (LMICs) (Iran/Nepal and India), while 3 originated from high income countries (HICs) (Spain, United States of America, and Turkey) (World Bank, 2021), and one study was a systematic review. Findings of this study are presented and discussed within the following themes: middle ear related symptoms, anatomical changes due to middle ear pathologies, middle ear pathologies based on acoustic immittance measure, and hearing loss with a conductive element.

### Middle ear related symptoms

Six out of eight studies indicated the occurrence of middle ear-related symptoms, which include otalgia and aural fullness. Among the symptoms, otalgia appears to be the most common type of middle ear-related symptom reported in these studies (Bhatta et al., 2021; Boroujeni et al., 2021; Fidan, 2020; Maharaj et al., 2020; Raad et al., 2021; Wanna et al., 2021). A multicentre study conducted by Bhatta et al. (2021) with 331 participants who were COVID-19 positive reported an incidence of 1.8% for otalgia and 11.4% for aural fullness, while Raad et al. (2021) found that six out of eight participants presented with otalgia. While some studies have not explained the occurrence of otologic symptoms such as otalgia and aural fullness, which may be linked with the presence of middle ear pathologies at times (Stach & Ramachandran, 2022), Bhatta et al. (2021) suspect that these symptoms may be because of the inflammation in the nasopharynx, resulting from dysfunctional eustachian tubes.

While the incidence of otologic symptoms such as otalgia and aural fullness seems to be varied in these studies because of different sample sizes within the studies reviewed, their occurrence highlights the need for further investigation and proper documentation in order to prioritise and implement early identification and intervention in patients infected with COVID-19. Kreisman et al. (2014) report that otalgia and sense of fullness may result from middle ear pathologies such as otitis media or otitis externa. Stach and Ramachandran (2022) also reported that otologic symptoms such as otalgia and aural fullness may suggest the presence of auditory disorders such as middle ear pathologies and warrant a referral to a physician for further investigation and management. Therefore, otalgia and aural fullness must be considered as possible symptoms when a patient presents with COVID-19. In fact, Boroujeni et al. (2021) recommend that COVID-19 must also be suspected where patients’ only symptom is otalgia.

### Anatomical changes because of middle ear structures

Middle ear pathologies can change the anatomical structure(s) of the middle ear system, particularly the tympanic membrane (Møller, 2013). In this review, three studies (Dharmarajan et al., 2021; Fidan et al., 2020; Wanna et al., 2020) have reported on the anatomical changes, particularly of the tympanic membranes, in patients infected with COVID-19. Fidan et al. (2020) reported hyperaemia and bulging of the tympanic membrane, while in a case series by Dharmarajan et al. (2021), four participants presented with retracted tympanic membrane and two had dull tympanic membranes. While the third study (Wanna et al., 2020) did not specifically report on the anatomical structure of the tympanic membrane, this case study reported that a participant presented with purulent otorrhea, which may be indicative of tympanic membrane perforation (Ali & Alshareda, 2017).

Although structural changes of the tympanic membrane indicate the presence of middle ear pathologies, the presence of otorrhea, which indicates chronic middle ear pathologies, is difficult to attribute to COVID-19. Chronic middle ear pathologies are results of longstanding pathologies that were left untreated. Given that the study that reported on the purulent otorrhea was a case study (Wanna et al., 2020), further research with a large sample size is required to explore the presence of chronic otitis media in patients infected with COVID-19. Despite this, COVID-19 seems to contribute to middle ear pathologies that affect the tympanic membrane. This calls for hearing health professionals to be cognisant of the possibilities of structural changes in the tympanic membrane because of middle ear pathologies.

Given that some structural changes may not necessarily affect the mobility of the tympanic membrane and hearing, but if left untreated may become chronic (WHO, 2018), causing complications, it is crucial that hearing health professionals remain vigilant to this possibility. The use of measures that have high sensitivity and specificity in identifying these structural changes is important. The use of video otoscopy is recommended to clearly visualise structural changes in the tympanic membrane and make appropriate referrals (Sebothoma & Khoza-Shangase, 2018). This measure is also appropriate as it allows for remote assessment via tele-audiology (Khoza-Shangase, Moroe, & Neille, 2021).

### Middle ear pathologies based on acoustic immittance measure

Three of the studies reviewed (Bhatta et al., 2021; Boroujeni et al., 2021; Fidan, 2020) measured acoustic immittance to determine the presence or absence of middle ear pathologies. All these studies reported on tympanometry, while only one study (Boroujeni et al., 2021) presented on acoustic reflex thresholds as well. Type B tympanogram was the common type of tympanogram measured, with three studies indicating its presence in participants infected with COVID-19 (Bhatta et al., 2021; Boroujeni et al., 2021; Fidan, 2020). Bhatta et al. (2021) reported an incidence of 5.1% people presenting with type B tympanogram, with only 1.15% presenting with type C tympanogram. These studies provide crucial information about the presence of middle ear pathologies that affect the mobility of the tympanic membrane. Given that some hearing health professionals do not always include acoustic immittance measures such as tympanometry in their test battery (Emanuel, Henson, & Knapp, 2012; Sebothoma & Khoza-Shangase, 2021), this review indicates that these measures must be included, especially when assessing patients presenting with COVID-19.

While these studies have provided some crucial information about the mechano-acoustic characteristics of the middle ear, it is worth noting that this information is limited. Two of these studies were case studies (Boroujeni et al., 2021; Fidan, 2020) with single participants, with only one study utilising a multicentre design (Bhatta et al., 2021). Therefore, future studies with large sample sizes and use of methodologies that allow for comparison of participants with and without COVID-19 are warranted. Furthermore, all studies have used tympanometry with single probe tone to measure the presence or absence of middle ear pathologies. Given the low sensitivity and specificity of tympanometry with single probe tone (Kaf, 2011), future studies need to incorporate sensitive measures of middle ear function such as the wideband acoustic immittance (WAI) (Robinson, Thompson, & Allen, 2016; Sebothoma, Khoza-Shangase, Mol, & Masege, 2021).

### Hearing loss with a conductive element

A total of six studies (Bhatta et al., 2021; Boroujeni et al., 2021; Dharmarajan et al., 2021; Fidan, 2020; Maharaj et al., 2020; Raad et al., 2021) indicated the occurrence of hearing loss with a conductive element. Bhatta et al. (2021) reported the occurrence of mild conductive hearing loss (CHL) in 3.2% participants infected with COVID-19. While the other studies reported the occurrence of CHL, two of those studies did not report on the severity of the CHL. Only the study by Boroujeni et al. (2021) indicated that the severity of CHL in a participant infected with COVID-19 was mild to moderately severe. The occurrence of CHL in these studies seems to be because of the middle ear pathologies. In the studies by Bhatta et al. (2021) and Boroujeni et al. (2021), participants presented with abnormal tympanograms (e.g. type B), while in the other studies the presence of CHL seemed to co-occur with a type or a sequelae of middle ear pathology such as otitis media or tympanic membrane perforation.

As limited evidence exists in the studies reviewed, it is difficult to make a comparison of the proportion of CHL with studies from the general population or/and those with participants infected with other viruses such as the HIV. In the studies reviewed, three of the studies were case studies, with one being the systematic review. Despite the limited evidence from the reviewed studies, available evidence indicates the occurrence of CHL in this population, and therefore, highlights the need for further investigation of the possible hearing loss of this nature in this population. Møller (2013) reported that untreated CHL may cause the deprivation of sound into the auditory system and affect speech perception and communication. Therefore, assessment of CHL using appropriate test battery that will allow for early identification and intervention is crucial.

## Implications, limitations, and recommendations

This review provided some crucial information about middle ear function and pathologies in patients infected with COVID-19. Important implications for the study are raised. The study suggests that there is a need for continued audiological evaluation and possibly periodic monitoring for patients infected with COVID-19. Resource allocation that includes sensitive measures of middle ear pathologies is required, and initiatives to ensure that preventive audiological programmes are implemented and encouraged by current findings. There is a need for development of audiological guidelines that can be used with patients infected with COVID-19 as well as those not diagnosed with COVID-19 but presenting with new middle ear pathologies during the pandemic. Hearing health professionals also need to be aware of the risks of COVID-19 on middle ear function in order to implement effective preventive care, and to provide education and counselling to patients and their families. Finally, findings from this review raise implications for future research regarding COVID-19 and middle ear function, research that will assist clinicians with a comprehensive understanding of the association between COVID-19 and middle ear function. Such research should include longitudinal designs and must involve large sample sizes.

## Conclusion

This scoping review found limited studies on the effects of COVID-19 on middle ear function. However, the available research highlights that COVID-19 may potentially affect middle ear function. This is because COVID-19 has been found in the middle ear system of patients infected with the virus (Jeican et al., 2021). Furthermore, COVID-19 was shown to cause upper respiratory tract infection (Weiss & Navas-Martin, 2005), which can contribute to the development of middle ear pathologies. Findings of this research raise implications for future studies to use appropriate methodologies and to include large sample size. Trends or patterns of middle ear pathologies must also be established to determine the need for periodic monitoring if there is a need. Finally, although further investigation is warranted, hearing health professionals must be cognisant of the possibility of middle ear involvement in patients infected with COVID-19 and should incorporate sensitive measures of middle ear pathologies such as WAI in their audiological test battery.
